# *MT‐RNR1* genotype testing for preventing aminoglycoside‐mediated ototoxicity: A guideline developed by the UK Centre of Excellence in Regulatory Science and Innovation in Pharmacogenomics (CERSI‐PGx)

**DOI:** 10.1002/bcp.70712

**Published:** 2026-07-29

**Authors:** John H. McDermott, Sian Hilton, Cinzia Dello Russo, Nicola Booth, Simon Drysdale, Philip Howard, Stephen Hughes, Mallinath Chakraborty, Helen McDevitt, Charlotte Alston, Maya Desai, Mike Beadsworth, Adam Reynolds, Serim Min, Katherine Payne, Munir Pirmohamed, William G. Newman

**Affiliations:** ^1^ Division of Evolution, Infection and Genomics, School of Biological Sciences University of Manchester Manchester UK; ^2^ Manchester Centre for Genomic Medicine, St Mary's Hospital Manchester University NHS Foundation Trust Manchester UK; ^3^ Department of Pharmacology and Therapeutics, Institute of Systems, Molecular and Integrative Biology University of Liverpool Liverpool UK; ^4^ Department of Translational Medicine and Surgery, Section of Pharmacology Università Cattolica del Sacro Cuore – Fondazione Policlinico Universitario A. Gemelli, IRCCS Rome Italy; ^5^ Neonatal Intensive Care Unit, St Mary's Hospital Manchester University NHS Foundation Trust Manchester UK; ^6^ Oxford Vaccine Group, Children's Hospital University of Oxford Oxford UK; ^7^ NHS England and NHS Improvement – North East and Yorkshire, NHS England North‐East and Yorkshire Leeds UK; ^8^ Department of Pharmacy Chelsea and Westminster Hospital NHS Foundation Trust London UK; ^9^ Neonatal Medicine University Hospital of Wales Cardiff UK; ^10^ Neonatal Unit Royal Hospital for Children Glasgow UK; ^11^ Newcastle Highly Specialised Mitochondrial Diagnostic Service North East and Yorkshire Genomic Laboratory Hub Newcastle UK; ^12^ Department of Cystic Fibrosis and Respiratory Medicine Birmingham Women's and Children's Hospital Birmingham UK; ^13^ Liverpool School of Tropical Medicine Liverpool UK; ^14^ Specialist and Academic Medicine Royal Liverpool University Hospital Liverpool UK; ^15^ Royal Jubilee Maternity Hospital Belfast Health and Social Care Trust Belfast UK; ^16^ Manchester Centre for Health Economics, Division of Population Health, Health Services Research and Primary Care, School of Health Sciences University of Manchester Manchester UK; ^17^ The Wolfson Centre for Personalised Medicine, Centre for Drug Safety Science University of Liverpool Liverpool UK

**Keywords:** aminoglycoside, gentamicin, guideline, MT‐RNR1, ototoxicity, pharmacogenetic

## Abstract

Aminoglycosides are broad‐spectrum antibiotics used in the management of severe infections. Aminoglycosides are associated with nephrotoxicity and ototoxicity. Although dosing strategies such as once‐daily administration and therapeutic drug monitoring have reduced the incidence of nephrotoxicity, ototoxicity remains unpredictable and may occur at therapeutic concentrations. A strong association between specific mitochondrial DNA variants in *MT‐RNR1* (m.1555A > G, m.1494C > T and m.1095 T > C) and aminoglycoside‐induced hearing loss exists. These variants (frequency ~1 in 330 individuals across populations) predispose to irreversible, sensorineural hearing loss following aminoglycoside exposure, sometimes after a single dose. Avoidance of aminoglycosides is recommended at any detectable variant level. In England, laboratory‐based *MT‐RNR1* testing is nationally commissioned, whereas point‐of‐care testing in time‐critical settings like neonatal sepsis is delivered in some centres. Approximately 20% of aminoglycoside use is predictable providing opportunities for pre‐emptive pharmacogenetic testing. Where *MT‐RNR1* testing results are unavailable and clinical urgency is high, aminoglycoside treatment should not be delayed. Early health economic evidence suggests that point‐of‐care testing in neonates may be cost‐saving by preventing lifelong hearing loss. Regulatory and Health Technology Assessment bodies support targeted implementation of testing alongside further evidence generation. Overall, integration of *MT‐RNR1* pharmacogenetic testing offers a feasible and proportionate strategy to reduce harm while preserving access to life‐saving antibiotic therapy. This guideline is grounded in the latest evidence in this field but cannot account for all individual factors relevant to patient care. Therefore, prescribers must conduct a thorough assessment of each patient's risk–benefit profile, ensuring that therapy is optimized to maximize benefits while minimizing potential harms.

## 1 BACKGROUND AND OVERVIEW

Aminoglycosides are natural or semisynthetic antibiotics derived from the filamentous bacteria Actinomycetes. They were among the first antibiotics to be introduced for routine clinical use, and several examples have been approved for use in humans. Streptomycin was the first drug in class licenced for the treatment of tuberculosis in the 1940s. Following this, other members of the drug class, including neomycin, kanamycin, gentamicin, netilmicin, tobramycin and amikacin were developed.^1^ Even though they are effective antimicrobials, their side effect profiles and the need for therapeutic drug monitoring led to a shift towards alternative agents. With the increasing emergence of multidrug resistance, the use of aminoglycosides has increased, and recently novel aminoglycosides have been developed, including arbekacin and plazomicin.^1^

The side effect profiles are similar across the antibiotic class, with both nephrotoxicity and ototoxicity recognized complications, with up to 17% of individuals experiencing ototoxicity following intravenous administration of aminoglycosides.^2^, ^3^ Dosing regimens and therapeutic drug monitoring have been used to reduce aminoglycoside toxicity (especially nephrotoxicity) while maintaining their effectiveness. Now a once‐daily, high‐dose regimen approach is accepted as the preferred standard administration.^4^ However, ototoxicity remains unpredictable and may occur at therapeutic concentrations.

Aminoglycosides bind to the tRNA binding site of 16S ribosomal RNA in the 30S subunit of bacterial ribosomes, disrupting mRNA translation, causing accumulation of truncated and dysfunctional proteins, which kill sensitive bacteria.^5^ They are active against various Gram‐positive and Gram‐negative organisms, especially members of the Enterobacteriaceae family, including *Escherichia coli* and *Klebsiella pneumoniae*.^1^ The class has activity against *Staphylococcus aureus*, including methicillin‐resistant and vancomycin‐resistant strains. They are used to treat *Pseudomonas aeruginosa* and Mycobacterium species, including extensive drug‐resistant *Mycobacterium tuberculosis* in the management of non‐tuberculous mycobacterial infection, for example, *Mycobacterium avium* and *Mycobacterium abscessus*. This broad‐spectrum activity of aminoglycosides is enhanced through synergy with other classes of antimicrobials, especially beta‐lactams.^6^

In 1993, a variant m.1555A > G in the mitochondrial DNA encoded gene *MT‐RNR1* was associated with increased susceptibility to sensorineural hearing loss (SNHL) following exposure to aminoglycosides.^7^ Subsequent studies have identified additional variants in *MT‐RNR1* (m.1095 T > C and m.1494C > T), which also predispose to aminoglycoside induced hearing loss (AIHL). These variants are found at different frequencies across different biogeographical groups (Table 1).^8^ In combination, the frequency of these variants is approximately 1 in 330 individuals.

**TABLE 1:** Frequencies of *MT‐RNR1* alleles in biogeographical groups (%).^8^

MT‐RNR1 allele	Central/South Asian	East Asian	European	Near Eastern	Sub‐Saharan African
m.1095 T > C	0.067%	0.146%	0.057%	0.000%	0.000%
m.1494C > T	0.067%	0.000%	0.035%	0.000%	0.000%
m.1555A > G	0.111%	1.810%	0.113%	0.142%	0.300%

In England, laboratory‐based testing for the three *MT‐RNR1* variants is nationally commissioned and available on the National Genomic Test Directory^9^ for individuals who are likely to require aminoglycoside treatment or to investigate an explanation for SNHL in an individual previously exposed to aminoglycosides.

## 2 LICENCED INDICATIONS

As gentamicin is the most frequently used aminoglycoside in the United Kingdom,^4^ the information for this drug is presented in this guideline. Uses of the other members of this group licenced for use in the United Kingdom in humans are listed in Appendix S1.

**Table:** 

Current licenced indications for gentamicin in the UK^a^, ^10^
Gentamicin is indicated for the treatment of the following infections in adults and children from birth when less toxic antimicrobial agents are not effective. Under these conditions, gentamicin is indicated in the treatment of the following infections: Complicated urinary tract infection (cUTI), including acute pyelonephritis Complicated intra‐abdominal infection (cIAI) Hospital‐acquired pneumonia (HAP) including ventilator‐associated pneumonia (VAP) Broncho‐pulmonary infections in patients with cystic fibrosis Infective endocarditis Burn wound infections Treatment of patients with bacteraemia that occurs in association with, or is suspected to be associated with, any of the infections listed above. Gentamicin should for all indications, except complicated urinary tract infections, only be used in combination with other relevant antibiotics (predominantly together with a beta‐lactam antibiotic or with an antibiotic effective against anaerobic bacteria).

^a^ The exact wording for the currently licenced indications has been extracted from the Summary of Product Characteristics (SmPC) for gentamicin 40 mg/ml solution for injection/infusion, Panpharma UK Ltd, approved by the Medicines and Healthcare products Regulatory Agency (MHRA, text revised on 16 October 2025).^10^ Note that each SmPC is specific to a product (including formulation and strength) and not to gentamicin.

In addition to the SmPC extract above detailing licenced indications, it should also be noted that NICE guidance NG195 recommends treatment for suspected early‐onset neonatal sepsis with a combination of intravenous benzylpenicillin and gentamicin.^11^ In paediatric oncology settings, gentamicin is commonly used when sepsis is suspected.

Gentamicin should be given as a single daily dose (in neonates, it can be every 36 h), with therapeutic drug monitoring (measuring trough levels) before further doses, to balance effective treatment against the risk of toxicity. The prescribing of aminoglycosides has changed over time due to the availability of new evidence that has led to changes in clinical practice. For example, it is routine practice now to administer single doses of gentamicin in patients admitted acutely with an uncomplicated urinary tract infection or with suspected sepsis, where studies (both RCTs and observational studies) have shown benefits in terms of quicker symptom resolution or decreased risk of mortality with no increase in the risk of acute kidney injury.^12^, ^13^ Gentamicin is no longer the recommended drug for the treatment of *S. aureus* infective endocarditis which is the most common form of acute and destructive disease, except for *S. aureus* endocarditis in the context of a prosthetic valve, but it is still used to treat endocarditis due to other causes.^14^

More broadly, aminoglycosides are recommended for the treatment of non‐tuberculous mycobacterial pulmonary disease as per the guidelines from the British Thoracic Society^15^ and the American Thoracic Society.^16^

## 3 EVIDENCE OVERVIEW

Multiple studies have reported an association between variants in *MT*‐*RNR1* and AIHL.^7^, ^8^, ^17^ These studies have mostly been small and retrospective and without extensive control data from healthy populations. However, systematic reviews have demonstrated that this association is robust especially for m.1555A > G and c.1494C > T with less compelling but strong evidence for m.1095 T > C.^8^, ^17^

Most studies have been undertaken in China with many different study designs having been used. Genotyping approaches have also been variable with the inclusion of different *MT*‐*RNR1* variants.

Penetrance (development of SNHL in individuals harbouring a pathogenic *MT‐RNR1* variant following exposure to an aminoglycoside) has been estimated at 63–100% for the m.1555A > G variant but is lower for c.1494C > T, ranging from 21–40%.^17^

Previous genetic testing technologies were less sensitive, which meant that the outcome of *MT*‐*RNR1* variant testing was binary and considered to be either present or absent. Therefore, when the m.1555A > G variant was detected, it was reported as homoplasmic, that is, present in all mitochondria in all cells of the body. It is known that mitochondrial variants can be present in some but not other mitochondrial genomes (a phenomenon termed heteroplasmy). The specific level of heteroplasmy at which a mitochondrial genetic variant is clinically relevant is not clear, but one early study found that individuals with a m.1555A > G heteroplasmy level <20% were consistently asymptomatic on exposure to aminoglycosides, whereas those with a level >50% developed ototoxicity.^18^ Most genotyping approaches employed now will detect heteroplasmy at levels of 10% or lower.

Outside of the *MT*‐*RNR1* association with AIHL, a study reported that the m.1555A > G variant is associated with SNHL irrespective of exposure to aminoglycosides.^19^ However, this relationship has not been supported by large population‐based cohort studies.^20^

## 4 RECOMMENDED INDICATIONS FOR PHARMACOGENETIC TESTING

Most studies have considered the risk of AIHL in the context of the use of aminoglycosides in neonates and children, with gentamicin being the most studied. There is some evidence that the risk of hearing loss is greatest in neonates (perhaps due to immaturity of the inner hair cells in the cochlea), but the risk of AIHL is also significant in healthy young people, for example, when aminoglycosides are used in women with infection following delivery of a child. However, older individuals are already more likely to have hearing impairment and the risk of additional impairment secondary to AIHL would suggest that the recommendation for testing should not be limited to particular age groups.

AIHL has been reported after a single dose of an aminoglycoside in susceptible individuals,^21^ and so pharmacogenetic testing is indicated before the first administration of an aminoglycoside, if this is clinically feasible.

The frequency of the three variants in *MT‐RNR1* across all biogeographical groups suggests that pharmacogenetic testing should be offered to all individuals, irrespective of genetic ancestry and prior to aminoglycoside treatment.

Aminoglycosides are often prescribed as combination antimicrobial therapy, for example, in adults with sepsis of unknown origin and NICE (guideline NG195)^11^ recommends treatment of sepsis or suspected sepsis in neonates with a combination of gentamicin and a beta‐lactam (benzylpenicillin). The recommendation for pharmacogenetic testing should not be different in the context of co‐medication.

There is insufficient evidence to suggest that the risk of AIHL is only confined to some aminoglycosides. As such, it is considered that the risk is relevant across all aminoglycosides and so pharmacogenetic testing should be undertaken prior to aminoglycoside administration where this is feasible and available.

In most instances, pharmacogenetic data will not be available at the point of decision to treat. In cases where the clinical risk is severe and aminoglycosides are the preferred treatment, use of aminoglycosides should not be delayed, and administration should proceed according to guidelines. If a point‐of‐care test is available, then this should be considered.

Inhaled and nebulised administration of aminoglycosides, for example, nebulised liposomal amikacin licenced for the treatment of refractory *M. avium* complex pulmonary disease and inhaled tobramycin for the management of chronic pulmonary infection due to *P. aeruginosa* in patients with cystic fibrosis are associated with significant systemic exposure and AIHL has been described, usually in the presence of renal dysfunction,^22^, ^23^, ^24^, ^25^, ^26^, ^27^, ^28^, ^29^ and so pharmacogenetic testing of *MT‐RNR1* variants is warranted. Aminoglycosides are highly bioavailable by intramuscular, intravenous and intraperitoneal administration, and so pharmacogenetic testing should be undertaken in all individuals receiving the drug by these routes.^30^, ^31^ Ototoxicity has been reported following use of orthopaedic cement containing aminoglycosides, and avoidance of use may be considered in patients with increased risk *MT‐RNR1* variants.^32^ There are also reports of hearing loss after intrathecal or intraventricular administration of gentamicin and streptomycin, but the association with *MT‐RNR1* variants in conferring the risk of AIHL in this setting is unknown.^33^

As in all patients, topical otic use (administration of medication into the ear canal) of an aminoglycoside is contraindicated in the presence of a perforated tympanic membrane because of the high risk of ototoxicity, but the association with *MT‐RNR1* variants is unknown.

In contrast to these routes, enteral and topical administration of aminoglycosides typically has absolute bioavailability <1% and so is unlikely to cause AIHL, even in individuals with *MT*‐*RNR1* variants.^34^ Absorption of aminoglycosides from the gastrointestinal tract may occur in the presence of severe epithelial damage, such as in patients with severe inflammation, so pharmacogenetic testing would be appropriate if available in this setting.^35^, ^36^

Testing of *MT‐RNR1* variants is recommended as a Level 1 investigation in all children with progressive permanent SNHL, according to the British Association of Audiovestibular Physicians guidance 2018.^37^

In summary, pharmacogenetic testing of variants in *MT‐RNR1* should be undertaken in all individuals, of all age groups where this is possible in a clinically achievable time frame prior to use for all aminoglycosides and for all routes of administration, except topical or enteral (unless absorption is expected to be clinically significant).

## 5 INTEGRATING PHARMACOGENETIC TESTING INTO EXISTING CLINICAL PATHWAYS

As seen in the licenced indications section, gentamicin and other aminoglycosides can be used for the treatment of many infections in many different clinical settings. The use of aminoglycosides can be planned or predicted in about 20% of individuals. These clinical scenarios include the following:

- individuals undergoing an invasive procedure (testing could be planned at a pre‐surgery assessment clinic).
- individuals who are susceptible to infection, for example, individuals with cystic fibrosis (routinely as a reflex test following a positive genetic diagnosis), bronchiectasis or long‐term urinary catheter use.
- individuals with multi‐drug‐resistant tuberculosis (MDR‐TB) where the use of an aminoglycoside is being considered.
- individuals with non‐tuberculous mycobacterial infection, for example, *M. avium* and *M. abscessus*.

In England, the nationally commissioned test in the National Genomic Test Directory^9^ from NHSE should be requested. The results should be recorded in the patient record (ideally in the electronic record in a format that is immediately available to inform future prescribing decisions).

Usually, aminoglycosides are used in an acute clinical setting for the treatment of sepsis (or suspected sepsis). If pharmacogenetic testing information for *MT*‐*RNR1* is already available and has been recorded in the patient record, this should inform the antibiotic prescribing decision. In most individuals, however, this pharmacogenetic information will not be available and a point of care test will not be available. Aminoglycoside treatment should not be delayed, and administration should proceed according to local microbiological guidance, as the benefit exceeds the risk.

Pharmacogenetic testing should be considered for individuals with hearing loss who have been exposed to aminoglycosides.^9^ In many countries, there are existing guidelines to support audiologists to investigate paediatric onset hearing loss. For example. The British Association of Audiological Physicians (BAAP) have produced guidelines for the aetiological assessment of bilateral sensorineural hearing loss in children, with *MT‐RNR1* testing recommended where exposure to aminoglycosides is documented.^38^, ^39^ Patients with known AIHL *MT‐RNR1* variants or a family history of ototoxicity are advised to inform their doctor or pharmacist before they take an aminoglycoside.^40^

In some neonatal units, a *MT*‐*RNR1* point of care assay may be available, which can generate a result from a buccal swab in minutes to inform aminoglycoside prescribing.^41^ Where such testing is available and undertaken, the relevant clinical staff should be trained in the operation of the point of care platform and the result of the test used to inform prescribing decisions. Further details on point of care testing, are provided in Section 10.4.

## 6 WHICH GENE(S), VARIANTS, TURNAROUND TIME?

The *MT*‐*RNR1* variants m.1555A > G (NC_012920.1:g.1555A > G); m.1494C > T (NC_012920.1:g.1494C > T); m.1095 T > C (NC_012920.1:g.1095 T > C) are included in the NHS England National Genomic Test Directory^9^ and have evidence of an association with AIHL (refer to Appendix S2). As of February 2026, the NHSE National Genomic Test Directory sets a turnaround time for this test of 6 weeks. All seven NHSE Genomic Lab Hubs undertaking this test are accredited by the United Kingdom Accreditation Service (UKAS).

In an acute setting, for example, suspected sepsis or sepsis in a neonate, when the decision to use an aminoglycoside is time‐sensitive, a point of care test is an option to determine if a baby is at increased risk of AIHL. Ideally, this test should assay the same *MT*‐*RNR1* variants tested as part of the commissioned laboratory test. At present, in the United Kingdom, there is a single UKCA approved test (Genedrive MT‐RNR1 ID Kit), which tests for the m.1555A > G variant, which has been considered in a NICE Early Value Assessment [HTG665].^42^ This is a qualitative in vitro molecular diagnostic test, which can generate a result from a buccal swab in 26 min.^41^ All units undertaking this testing should co‐ordinate their testing with their local governance processes and be part of a quality assurance scheme to ensure the analytical validity of the testing.

### 6.1 Communication between primary care and specialists

The use of aminoglycosides largely occurs in hospitals. However, it is still important that teams in primary care are made aware of the results for testing for *MT‐RNR1* variants. Relevant SNOMED‐CT codes should be used to record results in both primary care and hospital care records, as follows:

### 6.2 Reporting test results

- At increased risk of aminoglycoside‐induced hearing loss due to mitochondrially encoded 12S ribosomal ribonucleic acid genotype (finding) SCTID: 4055281000000113.
- At normal risk of aminoglycoside‐induced hearing loss based on mitochondrially encoded 12S ribosomal ribonucleic acid genotype (finding) SCTID: 4055331000000115.

### 6.3 Procedural code

- Mitochondrially encoded 12S ribosomal ribonucleic acid variant analysis (procedure) SCTID: 2365491000000108.

Although *MT*‐*RNR1* associated AIHL is not an allergy, it is appropriate to record this as a drug reaction in the allergy section in the medical record. At present, this is the safest way to ensure that an individual with a *MT*‐*RNR1* variant is not prescribed an aminoglycoside at a future timepoint.

## 7 CLINICAL ACTIONS BASED ON GENOTYPE

Our recommendation is that aminoglycosides (unless administered topically or enterally; see Section 4) are to be avoided when an AIHL associated variant in *MT*‐*RNR1* is identified. An alternative antibiotic should be chosen based on local guidance informed by the microbiology team.

In the rare situation that an aminoglycoside is needed in an individual with a *MT*‐*RNR1* AIHL associated variant (i.e., when an alternative medication is not as effective), the clinical reasoning must be clearly documented and discussed with the patient/parent and with the local infectious disease/microbiology team.^8^

The absence of a *MT*‐*RNR1* AIHL associated variant does not eliminate the risk of AIHL. This is because not all tests assess all relevant *MT‐RNR1* variants, and there may be other genetic determinants of AIHL. Further, aminoglycosides can cause hearing loss through other mechanisms, associated with prematurity, renal impairment, severe inflammatory response syndrome, prolonged exposure and high plasma concentrations.

Guidance to avoid aminoglycosides at any level of heteroplasmy for an *MT‐RNR1* variant associated with AIHL is appropriate given that the levels of heteroplasmy differ between different tissues and the DNA from blood, saliva, or buccal cells may not accurately reflect the levels in the inner hair cells in the cochlea (which cannot be directly measured).

If an individual positive for a *MT*‐*RNR1* variant associated with AIHL has previously received aminoglycosides and not developed hearing loss, this does not abolish the risk from subsequent doses. Therefore, aminoglycosides should be avoided, if possible, in this situation and an alternative antibiotic prescribed based on local microbiology guidance. Audiological follow‐up should then be arranged for the patient carrying the *MT*‐*RNR1* variant who has been exposed.

If in a setting where point of care testing is routinely delivered for *MT‐RNR1* variants and the test fails, then if an antibiotic is required before a repeat test can be undertaken, the antibiotic pathway (as dictated by local microbiology guidance) should be used. Further details regarding point of care testing, are provided in Section 10.4.

Some vaccines contain trace amounts of aminoglycosides used as an excipient in their manufacture. It is recommended that the benefits of vaccination far outweigh any theoretical risks of ototoxicity from vaccination in an individual with an AIHL associated *MT‐RNR1* variant.^43^

## 8 OTHER PHARMACOGENETICS GUIDELINES

In this section, prescribing recommendations provided by other well established international pharmacogenetics consortia are referenced. Note, these are subject to update, and the original source should be referenced.

### 8.1 The clinical pharmacogenetics implementation consortium (CPIC) guideline^8^

The guideline strongly recommends avoiding aminoglycosides in individuals with a known *MT‐RNR1* variant associated with AIHL unless no effective alternatives exist, with use then limited to the shortest possible duration under expert supervision. It states that the integration of genotype data into clinical workflows can reduce preventable lifelong hearing loss without compromising infection management.

### 8.2 Specialist pharmacy service guidance on implementing pharmacogenomic testing for aminoglycosides^44^

This guidance highlights three variants (m.1555A > G, m.1095 T > C and m.1494C > T) in increasing the risk of ototoxicity after exposure to the aminoglycosides, amikacin, gentamicin, neomycin, and tobramycin. In England, it recommends testing via the National Genomic Test Directory but also mentions the use of point‐of‐care testing for neonates. The standard turnaround time is quoted as 6 weeks.

### 8.3 Dutch pharmacogenetics working group guideline

At the time of writing, there is no guideline relating to *MT‐RNR1* variants and aminoglycoside use from the Dutch Pharmacogenetics Working Group.

## 9 ECONOMIC EVALUATION

Two economic evaluations of *MT‐RNR1* m.1555A > G variant testing of aminoglycosides to identify the risk of ototoxicity were identified from a systematic search (conducted January 2026). One study came from authors based in the United States^45^ and one from the United Kingdom.^42^, ^46^ The target populations for each analysis differed, with one study focusing on children with cystic fibrosis^45^ and the other study on newborns requiring treatment for suspected neonatal infection.^42^, ^46^

### 9.1 Cystic fibrosis

Veenstra et al.^45^ assessed the cost‐effectiveness of screening for the m.1555A > G variant in a hypothetical cohort of children with cystic fibrosis, with an estimated mean age of first exposure to intravenous tobramycin of approximately 12 years. The analysis was stated to be from a societal perspective in the United States using a decision‐analytic model with a lifetime horizon. Genetic testing for the m.1555A > G variant was used to guide whether the aminoglycoside, intravenous tobramycin, could be used safely. If genetic testing indicated a risk of ototoxicity with intravenous tobramycin, then alternative antibiotics (a β‐lactam and a quinolone) in variant‐positive patients were recommended. This approach was compared with standard care, in which all patients received aminoglycoside‐based therapy without testing. The following costs (US$; 2006 price year) were included: direct medical costs and longer term costs associated with cochlear implantation (US$, 2006 price year). This approach is not consistent with the stated societal perspective. The analysis assumed a test cost of US$345 (~£270).

In the base‐case analysis, testing was assumed to reduce the incidence of severe aminoglycoside‐induced hearing loss by 0.12% and estimated to increase quality‐adjusted life expectancy by 0.0043 QALYs per person at an additional cost of US$338 per person, resulting in an incremental cost‐effectiveness ratio (ICER) of US$79300 per QALY gained. The results suggested that genetic testing in this context was not cost‐effective relative to the commonly cited threshold of $50 000 per QALY in the United States. Results were highly sensitive to several key assumptions. In particular, uncertainty in the estimate of mortality attributable to avoiding aminoglycoside use, with small increases in mortality outweighing the benefits of preventing hearing loss and leading to a net loss in QALYs compared with standard care. Overall, the study concluded that genetic testing was unlikely to be cost‐effective and could potentially lead to worse patient outcomes due to avoidance of the more effective first‐line antibiotic therapy.

### 9.2 Neonatal infection

A NICE Early Value Assessment (EVA) assessed the cost‐effectiveness of the point‐of‐care Genedrive MT‐RNR1 ID Kit in newborns.^42^, ^46^ The population was defined as neonates who need antibiotic treatment or who are anticipated to need antibiotic treatment and who are being considered for treatment with aminoglycosides. This early decision‐analytic cost‐effectiveness analysis was informed primarily by data from the PALOH study and compared the Genedrive MT‐RNR1 ID Kit to inform whether gentamicin was an appropriate antibiotic with standard care without prior testing.^41^, ^47^ If a positive test was returned, then alternative antibiotics would be prescribed, with cefotaxime used as the representative alternative in the early analysis. The analysis adopted an NHS England perspective and considered costs (£; 2021/2022 price year) and outcomes over the subsequent lifetime of the neonate. The analysis included direct healthcare costs associated with testing and long‐term hearing‐related care, whereas antibiotic costs were excluded due to their minimal impact on the results. The analysis assumed a cost of £130 for the Genedrive MT‐RNR1 ID Kit. The study suggested that genetic testing was potentially dominant, being both less costly and more effective than standard care, with an incremental cost saving of £58 per person and an incremental gain of 0.01 QALYs per person.^46^ The observed cost savings and health gains were primarily driven by the avoidance of lifelong hearing loss and its associated long‐term healthcare costs. In addition, the use of a contemporary point‐of‐care test with a relatively low price further contributed to the favourable cost‐effectiveness observed in the NICE EVA. There was substantial uncertainty highlighted in this early analysis, and the conclusions were that more evidence was required on the performance of the genetic testing, with test sensitivity emerging as a key driver.

## 10 REGULATORY CONSIDERATIONS

### 10.1 Gentamicin SmPC^10^ and MT‐RNR1

The gentamicin SmPC referenced in this guideline includes a specific paragraph on ototoxicity in Section 4. Special warnings and precautions for use. The SmPC highlights the increased risk of ototoxicity in patients with the m.1555A > G variant in the 12S rRNA gene. It notes that ototoxicity may occur even if aminoglycoside serum levels are within the recommended range during treatment and recommends the use of alternative treatments in these patients. Moreover, in patients with a maternal history of relevant mitochondrial variants or aminoglycoside induced deafness, alternative treatments or genetic testing prior to administration should be considered.

### 10.2 FDA label for gentamicin and MT‐RNR1^48^

The FDA label also highlights the importance of the m.1555A > G variant in predisposing to ototoxicity with gentamicin. It also warns that a history of maternal ototoxicity due to aminoglycosides should raise suspicion of this variant. The FDA recommends avoidance of aminoglycosides unless there are no alternatives and the benefit exceeds the risk of hearing loss.

### 10.3 MHRA 2021 drug safety update^40^

The MHRA issued a drug safety update in 2021 highlighting the increased risk of deafness in patients with mitochondrial variants receiving aminoglycosides (gentamicin, amikacin, tobramycin and neomycin). The update also stated that the increased risk of aminoglycoside‐associated ototoxicity in patients with certain mitochondrial variants was seen in patients where the aminoglycoside serum levels were within the recommended range. These mitochondrial variants are rare, and penetrance is uncertain. Genetic testing should not delay urgently needed aminoglycoside treatment but may be considered, especially before the start of recurrent or long‐term treatment. If an individual is suspected to have suffered from aminoglycoside‐induced hearing loss, this should be reported to the MHRA on a yellow card,^49^ irrespective of whether they have had a genetic test or not.

### 10.4 NICE early value assessment [HTG665]^42^

In 2023, NICE published an early value assessment guideline considering the use of point of care testing in an acute neonatal setting in the context of sepsis or suspected sepsis. NICE made the following recommendations:

1. The Genedrive MT‐RNR1 ID Kit can be used, whereas further evidence is generated as an option for detecting the genetic variant m.1555A > G to guide antibiotic (aminoglycoside) use and prevent hearing loss in newborns who are being considered for treatment with aminoglycosides.
2. Healthcare professionals should tell parents about the possible implications of positive test results for their baby and their family at an appropriate time and give support and information.
3. Positive point‐of‐care results should be confirmed by laboratory testing.

The recommendation is conditional on further evidence being generated on the following:

- how the test affects time to administering antibiotics
- how the test result affects antibiotic prescribing decisions
- the technical performance and accuracy of the test.

The PALOH‐UK study (ISRCTN10216938) is addressing the evidence gaps and is expected to be completed and published in late 2026 with progression to a full assessment as part of the NICE Diagnostics Assessment Programme.

## 11 OTHER CONSIDERATIONS

Apart from AIHL, there are no other known health‐implications of a *MT‐RNR1* variant that require pretest counselling.

As *MT‐RNR1* variants are inherited in a matrilineal manner; that is, the mother of a child with a homoplasmic *MT*‐*RNR1* variant associated with AIHL will also carry that variant and will thus be at risk of hearing loss if exposed to an aminoglycoside. The risks extend to all the child's siblings, the mother's siblings, the maternal grandmother and all the maternal aunts and uncles. This inheritance pattern can be explained through discussion following referral to a Clinical Genetics Department or using the resources available at GeNotes.^50^ Therefore, there is no need to undertake genetic testing to confirm the presence of the *MT‐RNR1* variant in these maternal relatives. Where the variant detected is heteroplasmic, testing in first degree maternal relatives can be considered.

Further, as *MT‐RNR1* variants are inherited in a matrilineal manner, as an alternative to point of care testing in neonates, there is an option to test all pregnant women for presence of a variant as this will reflect the genotype in all of her offspring.^51^ Challenges exist with this approach due to the economic impact of testing all pregnant women when aminoglycosides are indicated in less than 10% of all newborn children. An additional approach that has recently been considered is to generate MT‐RNR1 genotype data as part of noninvasive prenatal testing (NIPT).^52^ Studies are required to ensure that genetic information that is generated during pregnancy is available at the point of prescribing in a newborn to determine the effectiveness of these alternative approaches.

Testing of *MT‐RNR1* variants is currently not part of the UK newborn screening programme. As part of the Generation study,^53^ there is an opportunity to explore the value of this information when embedded in the child's electronic record so that aminoglycosides can be avoided in any child carrying a *MT‐RNR1* AIHL associated variant without requirement for further genomic testing.

## 12 RESEARCH RECOMMENDATIONS

Further research on the penetrance of *MT‐RNR1* variants associated with AIHL is required. This may be achieved through study of population cohorts (such as the Generation Study) and correlation with aminoglycoside use and linkage to medical records.

Research is required to determine the clinical utility of point of care *MT‐RNR1* testing prior to aminoglycosides in acute settings apart from in neonates.

Research to determine if testing mothers with a high‐risk pregnancy to infer *MT‐RNR1* genotypes in high‐risk births and neonates can provide an alternative approach to safe aminoglycoside prescription.

Further research is required to determine if other *MT‐RNR1* variants, for example, m.1005 T > C,^17^ are associated with AIHL.

In individuals with a *MT‐RNR1* variant who require an aminoglycoside, further research is required to determine the benefits of otoprotective agents.

Mechanistic studies to determine whether different aminoglycosides differ in their ability to cause deafness in the presence of *MT‐RNR1* variants should be conducted.

## AUTHOR CONTRIBUTIONS

John H. McDermott, Sian Hilton, Cinzia Dello Russo, Munir Pirmohamed and William G. Newman drafted the original guideline. Serim Min and Katherine Payne led the economic evaluation. All authors contributed to workshops to discuss the guidance, provided edits and approved the final version.

## CONFLICT OF INTEREST STATEMENT

The conflict‐of‐interest statements are documented in Table S1.

## Supporting information


**Table S1.** Competence, affiliations, and disclosure of conflicts of interest of the writing committee of the CERSI‐PGx Guideline for MT‐RNR1 genotyping and the prevention of Aminoglycoside‐mediated ototoxicity.
**Table S2.** Comments received during the consultation period by various organizations on the CERSI‐PGx Guideline for MT‐RNR1 Genotyping and the prevention of aminoglycoside‐mediated ototoxicity and Responses by the UK CERSI PGx writing committee.
Appendix S1. Licensed indications.
Appendix S2. NHSE National Genomic Test Directory Eligibility Criteria for *MT‐RNR1* variant testing.^9^.

## Data Availability

The data that support the findings of this guideline are publicly available, as referenced.
