# Supplementary material for: MT‐RNR1 genotype testing for preventing aminoglycoside‐mediated ototoxicity: A guideline developed by the UK Centre of Excellence in Regulatory Science and Innovation in Pharmacogenomics (CERSI‐PGx)
Source: Br J Clin Pharmacol. 2026 Jul 29;92(9):3002–11. doi: 10.1002/bcp.70712 (PMC13519768; doi:10.1002/bcp.70712)
Supplement: Supplementary file 1 [file BCP-92-3002-s001.docx]

***MT-RNR1* genotype testing for preventing aminoglycoside-mediated ototoxicity: A guideline developed by the UK Centre of Excellence in Regulatory Science and Innovation in Pharmacogenomics (CERSI-PGx)**

John H. McDermott^1,2^, Sian Hilton^2^, Cinzia Dello Russo^3,4^, Nicola Booth^5^, Simon Drysdale^6^, Philip Howard^7^, Stephen Hughes^8^, Mallinath Chakraborty^9^, Helen McDevitt^10^, Charlotte Alston^11^, Maya Desai^12^, Mike Beadsworth^13,14^, Adam Reynolds^15^, Serim Min^16^, Katherine Payne^16^, Munir Pirmohamed^3,17^, William G. Newman^1,2*^.

^1^Division of Evolution, Infection and Genomics, School of Biological Sciences, The University of Manchester, Manchester, UK.

^2^Manchester Centre for Genomic Medicine, St Mary’s Hospital, Manchester University NHS Foundation Trust, Manchester, UK.

^3^Department of Pharmacology and Therapeutics, Institute of Systems, Molecular and Integrative Biology, University of Liverpool, Liverpool, UK.

^4^Department of Translational Medicine and Surgery, Section of Pharmacology, Università Cattolica del Sacro Cuore - Fondazione Policlinico Universitario A. Gemelli, IRCCS, Rome, Italy

^5^Neonatal Intensive Care Unit, St Mary’s Hospital, Manchester University NHS Foundation Trust, Manchester, UK.

^6^Oxford Vaccine Group, Children's Hospital, University of Oxford, Oxford, UK.

^7^NHS England and NHS Improvement – North East and Yorkshire, NHS England North-East and Yorkshire, Leeds, UK.

^8^Department of Pharmacy, Chelsea and Westminster Hospital NHS Foundation Trust, London, UK

^9^Neonatal Medicine, University Hospital of Wales, Cardiff, UK.

^10^Neonatal Unit, Royal Hospital for Children, Glasgow, UK.

^11^Newcastle Highly Specialised Mitochondrial Diagnostic Service, North East and Yorkshire Genomic Laboratory Hub, Newcastle, UK.

^12^Department of Cystic Fibrosis and Respiratory Medicine, Birmingham Women's and Children's Hospital, Birmingham, UK.

^13^Liverpool School of Tropical Medicine, Liverpool, UK.

^14^Specialist and Academic Medicine, Royal Liverpool University Hospital, Liverpool, UK

^15^Royal Jubilee Maternity Hospital, Belfast Health and Social Care Trust, Belfast, UK.

^16^Manchester Centre for Health Economics, Division of Population Health, Health Services Research and Primary Care, School of Health Sciences, The University of Manchester, Manchester, UK.

^17^The Wolfson Centre for Personalised Medicine, Centre for Drug Safety Science, University of Liverpool, Liverpool, UK.

**Supplementary Methodology**

**Writing committee**

The UK CERSI-PGx guideline on *MT-RNR1* genotype testing for aminoglycosides was developed by a multidisciplinary team, including experts in clinical genetics, clinical pharmacology, pharmacology and pharmacogenomics, clinicians, including neonatologists and paediatricians, neonatal nurse, clinicians in paediatric infectious diseases, respiratory paediatrician, as well as experts in antimicrobial pharmacy, genomic medicine and health economics. The guideline committee included one member from Scotland, Wales and Northern Ireland in addition to members from England. The writing committee convened the first time online on 23 October 2025 and subsequently met online two times (on 14 November and 9 December) to finalise the guideline draft for consultation, with written contributions from members throughout this period. Details on the writing committee, as well as full disclosure of the affiliations, competences and conflict of interests is provided in Supplementary Table 1.

**Literature review**

Recommendations from the UK CERSI-PGx consortium are evidence-based together with expert input from committee members who were able to highlight nuances in specific clinical settings in which aminoglycosides are used. A comprehensive literature review was conducted on the *MT-RNR1* genetic variants and genotype testing for preventing aminoglycoside-mediated ototoxicity, integrating information provided by the latest CPIC guideline published in 2022^1^. We also consulted other guidelines/guidance produced by specialist societies and by NICE. In the Evidence Overview section, a summary of the most relevant evidence is provided. An extensive literature review was performed on specific topics, including the robustness of evidence for the three specific variants associated with AIHL as well as clinical relevance of heteroplasmy of the *MT-RNR1* variants. The section on Clinical actions based on genotype contains recommendations for avoiding aminoglycosides in patients with *MT-RNR1* AIHL-associated variants due to the risk of ototoxicity but where alternatives are ineffective, clinical reasoning should be documented. Whenever possible relevant UK prescribing guidelines have been quoted.

**Genetic variants to be tested**

The *MT-RNR1* genetic variants m.1555A>G, m.1494C>T and m.1095T>C are included in the NHS England National Genomic Test Directory and have been listed in this section. These variants have evidence of an association with AIHL and are included in the CPIC guideline for the use of aminoglycosides based on *MT-RNR1* genotype. The use of a point-of-care test in an acute setting is captured in this section if this is available and would ideally include all three *MT-RNR1* variants listed.

**Consultation process**

At the end of the writing process, the guideline final draft was shared in confidence for consultation with the Regulators (the MHRA, The National Institute for Health and Care Excellence), leading UK charities for hearing loss, Pharmacy Professional Networks, ClinPGx and the DPWG, Infection Control and Antimicrobial Management groups, like British Infection Association, Royal College of Paediatrics and Child Health, Neonatal & Paediatric Pharmacy Group and Association for Clinical Genomic Science, the Association of the British Pharmaceutical Industry, and the British in vitro Diagnostics Association. The consultation period started on 17 March 2026 and was open for one month, with a two-week extension to collect input from most stakeholders. Comments received are included in a single table together with the UK CERSI-PGx responses to each of the comments, and if appropriate, by changes in the guideline. The content of the table is published as Supplementary Table 2 to ensure transparency of the procedures undertaken.

**Patient And Public Involvement and Engagement (PPIE)**

The guideline was shared with three patient representatives, members of the PPIE group established for the delivery of work package 5 of the UK CERSI-PGx project^2^. A lay summary has been prepared with their contribution to explain the relevance of genetic variability in *MT-RNR1* in relation to aminoglycoside use. The lay summary is included in the supplementary material.

**Supplementary References**

1. McDermott JH, Wolf J, Hoshitsuki K, et al. Clinical Pharmacogenetics Implementation Consortium Guideline for the Use of Aminoglycosides Based on MT-RNR1 Genotype. *Clin Pharmacol Ther*. 2022; **111**(2): 366-372. doi:10.1002/cpt.2309
2. Pirmohamed M, Dello Russo C. UK Centre of Excellence in Regulatory Science and Innovation in Pharmacogenomics. *Br J Clin Pharmacol*. 2026; **92**(2): 327-328. doi:10.1002/bcp.70363

**Supplementary Plain English Summary**

Kamil Sterniczuk, Clair Conley and Catherine Lennon, members of the UK CERSI-PGx PPIE group.

**What are aminoglycosides?**

Aminoglycosides are antibiotics used to treat serious bacterial infections. They are commonly used in hospitals for conditions such as severe infections in the blood (sepsis), pneumonia, complicated urinary infections and infections in newborn babies. Examples include gentamicin, amikacin, and tobramycin. These medicines work by stopping bacteria from making the proteins that they need to survive and multiply. Although aminoglycosides are effective and often lifesaving, they can sometimes cause side effects. One of the most important risks is damage to hearing.

**Why do genes matter with aminoglycosides?**

In some people, aminoglycosides can cause hearing loss. This risk is strongly linked to certain genetic changes in a gene called *MT-RNR1*. There are three genetic variants linked to aminoglycoside-induced hearing loss, namely:

m.1555A>G

m.1494C>T

m.1095T>C

People who carry one of these variants are much more likely to develop hearing loss after receiving aminoglycosides. In some cases, hearing loss can occur suddenly even after a single dose. It can affect both ears and be permanent.

**Who is at higher risk?**

Not everyone who receives aminoglycosides develops hearing loss, but people who carry one of the *MT-RNR1* genetic variants are at increased risk. These variants are uncommon but not rare, affecting around 1 in 330 people. They can occur in people from many different populations. Because most people do not know whether they carry these variants, genetic testing is needed to identify those at risk.

**What do the prescribing guidelines say?**

The UK guideline recommends genetic testing for MT-RNR1 variants before aminoglycoside treatment whenever possible. Testing is particularly useful in situations where the need for aminoglycosides can be predicted in advance. For example, this may include people who are likely to require these antibiotics as part of planned treatment or ongoing care.

However, aminoglycosides are often used in urgent situations, such as suspected sepsis. In these cases, treatment should not be delayed while waiting for genetic test results. Rapid testing may sometimes be available to help guide treatment decisions in these settings.

**How do test results affect treatment?**

If a *MT-RNR1* genetic variant associated with hearing loss is present, aminoglycoside antibiotics should usually be avoided and an alternative antibiotic used. If no risk variants are found, aminoglycosides can usually be prescribed with greater confidence. However, hearing loss can still occasionally occur for other reasons, such as high doses, prolonged treatment, or other medical conditions.

**What does this mean for patients?**

Genetic testing helps doctors choose the safest treatment before antibiotics are given. This reduces the risk of permanent hearing loss while ensuring patients still receive effective treatment for serious infections. Preventing hearing damage is particularly important for babies and young children because hearing plays a key role in speech and language development. Test results should be recorded in medical records so healthcare professionals can consider them when prescribing antibiotics in the future.

**Key message**

Aminoglycosides are important antibiotics used to treat serious infections. However, some people carry genetic variants that greatly increase their risk of permanent hearing loss after receiving these medicines. A simple genetic test can identify those at risk before treatment begins. When a risk variant is found, alternative antibiotics can usually be used. This approach improves patient safety and supports more personalised and informed care.

**APPENDIX 1: LICENSED INDICATIONS**

**Current licensed indications for Amikacin in the UK^54^**

Amikacin Injection is a semi-synthetic, aminoglycoside antibiotic which is active against a broad spectrum of Gram-negative organisms, including pseudomonas and some Gram-positive organisms.

Sensitive Gram-negative organisms include; Pseudomonas aeruginosa, Escherichia coli., indole-positive and indole-negative Proteus spp., Klebsiella, Enterobacter and Serratia spp., Minea-Herralae, Citrobacter freundii, Salmonella, Shigella, Acinetobacter and Providencia spp.

Many strains of these Gram-negative organisms resistant to gentamicin and tobramycin show sensitivity to amikacin in vitro.

The principal Gram-positive organism sensitive to amikacin is Staphylococcus aureus, including some methicillin-resistant strains. Amikacin has some activity against other Gram-positive organisms including certain strains of Streptococcus pyogenes, Enterococci and Diplococcus pneumoniae.

Amikacin is indicated in the short-term treatment of serious infections due to susceptible strains of Gram-negative bacteria, including Pseudomonas species. Although amikacin is not the drug of choice for infections due to staphylococci, at times it may be indicated for the treatment of known or suspected staphylococcal disease. These situations include: the initiation of therapy for severe infections when the organisms suspected are either Gram-negative or staphylococci, patients allergic to other antibiotics, and mixed staphylococcal/Gram-negative infections.

Therapy with amikacin may be instituted prior to obtaining the results of sensitivity testing. Surgical procedures should be performed where indicated.

*	The exact wording for the currently licensed indications has been extracted from the Summary of Product Characteristics of Amikacin 250mg/ml injection, Hospira UK Ltd (EMC, text revised on 05/04/2024).^54^

In addition to these licensed indications, in patients with cystic fibrosis and other causes of bronchiectasis, amikacin (both iv and nebulised) is used in treating non-tuberculous mycobacterial infections.

**Current licensed indications for Tobramycin in the UK^55^**

Tobramycin Injection is indicated in the treatment of the following serious infections caused by susceptible micro-organisms:

• The treatment of central nervous system infections including meningitis, septicaemia and neonatal sepsis

• The treatment of gastro-intestinal infections including peritonitis

• The treatment of complicated and recurrent urinary tract infections such as pyelonephritis and cystitis

• The treatment of lower respiratory tract infections, including pneumonia, bronchopneumonia and acute bronchitis

• The treatment of skin, bone and soft tissue infections including burns.

Tobramycin may also be considered in serious staphylococcal infections for which penicillin or other less potentially toxic drugs are contraindicated and when bacterial susceptibility testing and clinical judgement indicate its use.

*	The exact wording for the currently licensed indications has been extracted from the Summary of Product Characteristics of Tobramycin, 40mg/ml Solution for Injection, Hospira UK Ltd (EMC, text revised on 29/02/2024).^55^

**Streptomycin is unlicensed in the UK**

Streptomycin is considered for use by deep intramuscular injection in individuals for *M. tuberculosis* resistant to other treatments when used in combination with other antituberculous antimicrobials.

With doxycycline for the treatment of brucellosis (on expert advice).

For treatment of enterococcal endocarditis.

**Current licensed indications for Neomycin**

Neomycin has licensed formulations for eye drops, ear drops and spray as well as ointments and creams (https://www.medicines.org.uk/emc), mostly as combination preparations

Oral administration for bowel decontamination prior to surgery or for patients in a hepatic coma

**Current licensed indications for Framycetin**

Bacterial infection in otitis externa

**Current licensed indications for Netilmicin**

Treatment of local eye infections

**APPENDIX 2: NHSE National Genomic Test Directory Eligibility Criteria for *MT-RNR1* variant testing.^9^**

The exact wording for the eligibility criteria has been extracted from the National Genomic Test Directory Version 9, published 8 April 2026. The test directory is updated annually, and the most current version should be referred to.

**Supplementary Table 1.** *Competence, affiliations, and disclosure of conflicts of interest of the writing committee of the CERSI-PGx Guideline for MT-RNR1 genotyping and the prevention of Aminoglycoside-mediated ototoxicity.*

| **Name** | **Main Expertise** | **Full Affiliation** | **Institution/s** | **Nation** | **Conflicts of Interest** |
| --- | --- | --- | --- | --- | --- |
| William Newman  CHAIR  Prof | Clinical Genetics  Pharmacogenomics | Professor of Translational Genomic Medicine and Director, Greater Manchester Genomic Medicine Centre | University of Manchester | England | Co-founder & CSO Fava Health  Clinical Advisory Board Illumina  Conference funding – Genedrive plc.  Awarded funding from NIHR-i4i and OLS for PALOH UK (Pharmacogenetics to Avoid Loss of Hearing (PALOH) trial UK; 2024-206) and by NIHR i4i (Development and Implementation of a Point-of-Care Pharmacogenetic Test to Avoid Antibiotic Related Hearing Loss in Neonates. PALOH; 2018-2021) with GeneDrive as partners. |
| Munir Pirmohamed  Prof | Clinical Pharmacology Internal Medicine  Pharmacogenomics | Department of Pharmacology and Therapeutics, Wolfson Centre for Personalised Medicine, University of Liverpool, Liverpool, UK.  Liverpool University Hospital Foundation NHS Trust, Liverpool, UK. | University of Liverpool | England | Currently receives partnership funding, paid to the University of Liverpool, for the MRC Medicines Development Fellowship Scheme (co-funded by MRC and GSK, AZ, Optum and Hammersmith Medicines Research). He has developed an HLA genotyping panel with MC Diagnostics but does not benefit financially from this. He is part of the IMI Consortium ARDAT ([www.ardat.org](http://www.ardat.org)); none of these funding sources have been used for the guideline |
| Cinzia  Dello Russo  Dr | Pharmacology  Pharmacogenomics | Department of Pharmacology and Therapeutics, Institute of Systems Molecular and Integrative Biology (ISMIB), University of Liverpool, Liverpool, UK.  Department of Translational Medicine and Surgery, Section of Pharmacology, Università Cattolica del Sacro Cuore - Fondazione Policlinico Universitario A. Gemelli, IRCCS, Rome, Italy | University of Liverpool  Università Cattolica del Sacro Cuore - Fondazione Policlinico Universitario A. Gemelli, IRCCS, Rome, Italy | England | No conflicts |
| John McDermott  Dr | Genomic Medicine | NIHR Academic Clinical Fellow in Genomic Medicine, Manchester University NHS Foundation Trust | University of Manchester | England | Co-founder of Fava Health  Honoraria for conference presentations – GeneDrive plc  Awarded funding from NIHR-i4i and OLS for PALOH UK (Pharmacogenetics to Avoid Loss of Hearing (PALOH) trial UK; 2024-206) and by NIHR i4i (Development and Implementation of a Point-of-Care Pharmacogenetic Test to Avoid Antibiotic Related Hearing Loss in Neonates. PALOH; 2018-2021) with GeneDrive as partners. |
| Nicola Booth  Dr | Neonatal Nurse | Research Nurse Manager/NICU  Newborn Intensive Care Unit (Ward 68)  St Mary’s Hospital  Oxford Road,  Manchester  M13 9WL | St Mary’s Hospital | England | No conflicts |
| Simon B Drysdale  Dr | Paediatric Infectious Diseases and Immunology | Associate Professor \| Oxford Vaccine Group, Department of Paedicatrics,  Consultant in Paediatric Infectious Diseases and Immunology \| Oxford  Co-Chair, Paediatric Allergy, Immunology and Infectious Diseases RCPCH College Speciality Advisory Committee and NIHR Oxford Biomedical Research Centre, Oxford UK  Deputy Director, PGCert, PGDip and MSc in Paediatric Infectious Diseases, University of Oxford | University of Oxford | England | SBD has provided consultancy and/or investigator roles in relation to product development for Janssen, AstraZeneca, Pfizer, Moderna, Valneva, GSK, MSD, iLiAD, Mundi Pharma and Sanofi with fees paid to his institution. SBD is a member of the UK Department of Health and Social Care’s (DHSC) Joint Committee on Vaccination and Immunisation (JCVI) RSV subcommittee and Medicines and Healthcare products Regulatory Agency’s (MHRA) Paediatric Medicine Expert Advisory Group (PMEAG). He is on the PALOH-UK study trial steering committee. |
| Philip Howard OBE  Prof |  | AMR Regional Antimicrobial Stewardship Lead, NHS England and NHS Improvement – North East and Yorkshire, UK and immediate Past President, BSAC | NHS England North-East and Yorkshire | England | No conflicts |
| Stephen Hughes  Dr | Antimicrobial Pharmacist | Consultant antimicrobial pharmacist, Chelsea and Westminster Hospital NHS Foundation Trust | Cheslea and Westminster Hospital NHS Foundation Trust | England | Consulted or provided educational support from Advanz, Baxter, Bowmed, Eumedica, Gilead, NAPP, Pfizer, Shionogi and Tillots |
| Mallinath Chakraborty  Dr | Neonatal Medicine | Consultant in Neonatal Medicine, University Hospital of Wales, Cardiff | University Hospital of Wales | Wales | No conflicts |
| Helen McDevitt  Dr | Neonatologist and Paediatrician | Consultant Neonatologist and paediatrician, Royal Hospital for children, Glasgow | Royal Hospital for Children, Glasgow | Scotland | Study site Principal Investigator for the PALOH-UK study |
| Charlotte Alston  Dr | Clinical Scientist | Principal Clinical Scientist \| NE&Y GLH Rare Disease Service Lead (Mitochondrial Disease) \| NIHR post-doctoral research fellow  Chief Investigator – The diagnosis of paediatric mitochondrial disease without muscle biopsy | Newcastle Highly Specialised Mitochondrial Diagnostic Service | England | No conflicts |
| Maya Desai  Dr | Respiratory Medicine | Consultant respiratory paediatrician with an interest in cystic fibrosis at Birmingham Children’s Hospital | Birmingham Children’s Hospital | England | No conflicts |
| Mike Beadsworth  Dr | Infectious Diseases | Hon Senior Lecturer Liverpool School of Tropical Medicine  Clinical Director Specialist and Academic Medicine RLUH  NHS consultant in infectious diseases | RLUH and LSTM | England | No conflicts |
| Adam Reynolds  Dr | Neonatology | Consultant Neonatologist  Royal Jubilee Maternity Hospital  Belfast Health and Social Care Trust | Belfast Health and Social Care Trust | Northern Ireland | No conflicts |
| Katherine Payne  Prof | Health Economics,  Pharmacy  Pharmacogenomics | Manchester Centre for Health Economics, Division of Population Health, Health Services Research and Primary Care, School of Health Sciences, The University of Manchester, Manchester, UK | University of Manchester | England | No conflicts |
| Sian Hilton  Ms | Project Management | Manchester Centre for Genomic Medicine, St Mary’s Hospital, Manchester University NHS Foundation Trust, Manchester, UK | Manchester University NHS Foundation Trust | England | No conflicts |
| Serim Min  Dr | Health Economics | Manchester Centre for Health Economics, Division of Population Health, Health Services Research and Primary Care, School of Health Sciences, The University of Manchester, Manchester, UK | University of Manchester | England | No conflicts |

**Supplementary Table 2.** Comments received during the consultation period by various organisations on the CERSI-PGx Guideline for MT-RNR1 Genotyping and the prevention of aminoglycoside-mediated ototoxicity and Responses by the UK CERSI PGx writing committee

| **Organisation** | **Comments from Organisation** | **Response from Committee** | **Changes to the guideline (if any)** |
| --- | --- | --- | --- |
| MHRA | **MHRA Comments - MT-RNR1 genotyping before the prescribing of Aminoglycosides**  This guideline represents a valuable and timely contribution to the development of evidence-based recommendations for individual drug–gene pair associations, with a clear focus on the use of MT-RNR1 genotyping prior to aminoglycoside prescribing. The multidisciplinary composition of the guideline development group strengthens its clinical relevance and applicability, particularly within the context of UK practice.  The sections addressing patient eligibility, the proposed clinical pathway, and recommended management strategies for individuals carrying MT-RNR1 risk variants are clearly structured and clinically meaningful. These elements are likely to support safe and effective implementation in routine care and help mitigate the risk of aminoglycoside-induced ototoxicity.  Overall, the guideline is of high quality and would provide significant clinical benefit. To further strengthen the document, it would be helpful to include details on the current extent of clinical use of MT-RNR1 genotyping and any available preliminary findings or implementation data to date. This would provide additional context regarding feasibility, uptake, and real-world impact.  Future considerations should include further discussion on recommended next steps in the event of a positive result in clinically urgent scenarios. Specifically, additional guidance on immediate prescribing decisions, alternative antibiotic strategies, and risk–benefit considerations in time-critical situations would support clinicians in applying the guideline effectively under acute care conditions.  Some additional considerations include:   1- This guideline refers to the point of care testing and recommends it for example in sections 5 and 6. However, the guideline does not refer to limitations of the point of care testing. It is only in section 10.2 that there is more information about NICE Early Value Assessment [HTE6] and that further evidence is being generated on the point of care test Genedrive MT‑RNR1 ID Kit etc. 2- The authors may consider also adding the reference for the NICE Early Value Assessment [HTE6]- furthermore according to the NICE website, [https://www.nice.org.uk/guidance/hte6-](https://eur01.safelinks.protection.outlook.com/?url=https%3A%2F%2Fwww.nice.org.uk%2Fguidance%2Fhte6-&data=05%7C02%7CShareen.Doak%40mhra.gov.uk%7Cf4f0c23772304adf30e208de9719a247%7Ce527ea5c62584cd2a27f8bd237ec4c26%7C0%7C0%7C639114334140970038%7CUnknown%7CTWFpbGZsb3d8eyJFbXB0eU1hcGkiOnRydWUsIlYiOiIwLjAuMDAwMCIsIlAiOiJXaW4zMiIsIkFOIjoiTWFpbCIsIldUIjoyfQ%3D%3D%7C0%7C%7C%7C&sdata=WrsMnhzeSNnacTQH9LA8domi8OWafJghnSGv5zHRsn0%3D&reserved=0) they “*have moved Health technology evaluation 6 to become* [*HealthTech guidance 665*](https://eur01.safelinks.protection.outlook.com/?url=https%3A%2F%2Fwww.nice.org.uk%2Fguidance%2Fhtg665&data=05%7C02%7CShareen.Doak%40mhra.gov.uk%7Cf4f0c23772304adf30e208de9719a247%7Ce527ea5c62584cd2a27f8bd237ec4c26%7C0%7C0%7C639114334140996950%7CUnknown%7CTWFpbGZsb3d8eyJFbXB0eU1hcGkiOnRydWUsIlYiOiIwLjAuMDAwMCIsIlAiOiJXaW4zMiIsIkFOIjoiTWFpbCIsIldUIjoyfQ%3D%3D%7C0%7C%7C%7C&sdata=pul9iUD2rAjuEeJooXCo62Yu6Q2K8vMnDUl9DLR3ORU%3D&reserved=0)*.- “* 3- Please also see suggestions in attached paper:   **Minor suggestions:**  **Section 8: *FDA Label for gentamicin and MT-RNR1:***  **MHRA Comment**: Suggest this is moved to section 10, Regulatory Considerations.  Could be replaced with: American College of Medical Genetics and Genomics (ACMG) 2014 guideline ("ACMG Practice Guideline for the Clinical Evaluation and Etiologic Diagnosis of Hearing Loss") supports that single-gene testing may be considered for individuals with a family history of aminoglycoside ototoxicity.  **Section *10.1 MHRA 2021 Drug Safety Update:***  **MHRA Comment**: If FDA product warnings are moved to this section, I would suggest also including MHRA product label warnings & testing recommendations here too  ***Section 10.2 NICE Early Value Assessment [HTE6]***  **MHRA Comment**: Suggest this is moved up to Section 8 ‘OTHER PHARMACOGENETICS GUIDELINES’ | We thank the MHRA for their positive comments.  Thank you for the positive comment. As mentioned in section 10.4, NICE recommended the use of point care testing in the acute neonatal setting and the feasibility of test implementation in the NHS has been shown in our recent publication (McDermott JH et al., *JAMA Pediatr*. 2022;176(5):486-492). Thus, as reported in section 5, this test is currently available in some neonatal units, including the Manchester University NHS Foundation Trust (MFT). However, the CERSI-PGx guideline extends the use of PGx testing for the MT-RNR1 risk variants to other indications as reported in Section 5. For these indications, the test can be requested via the National Genomic Test Directory according to specific eligibility criteria (as reported in Appendix 2).  In the context of a positive *MT-RNR1* variant result, we recommend referral to a clinical genetics service for appropriate counselling and refer to the GeNotes resource.  Uptake of R65 testing is a dynamic process and efforts are in place to capture this information and to encourage its appropriate use. Regarding point of care testing, the results of the PALOH-UK study will be published later in 2026.  As reported in Section 7, the strong recommendation by CERSI-PGx is to avoid the use of aminoglycosides in people carrying the *MT-RNR1* risk variants. We include a generic recommendation about alternative treatment regimens, since the choice will be informed by local guidance and these guidelines may change over time.  1 - Thank you for your comment. We have now included in sections 5 and 6 a link to section 10.4.  2 – We have amended the reference list.  3 – We have responded to the individual comments, below.  We have revised Section 8 and to move the paragraph on the FDA label for gentamicin and MT-RNR1 from Section 10 as suggested.  We have amended Section 10 according to this suggestion.  Thank you for your suggestion. We consider NICE guidelines as part of the UK regulatory framework thus this section was not changed. | No  No  No  No  Yes, sections 5 and 6  Yes, section 10 and reference list  Yes, sections 8 and 10  Yes, section 10  No |
| NICE | Thanks for sharing this. I don't think that we (NICE) really have any comments to make on the content, other than to say that it looks good. This will be really valuable for us when we start to produce guidance on the tests in the summer. | Thank you for the positive comments and support. |  |
| British Pharmacological Society | No response received |  |  |
| UK Clinical Pharmacy Association (UKCPA) | **Feedback to the CERSI-PGx ‘MT-RNR1 genotype testing for preventing aminoglycoside-mediated ototoxicity’ guidance, from the UKCPA committees (UKCPA Genomics Committee and members including GMS Lead pharmacists and GMS senior pharmacists, UKCPA Critical Care Committee, UKCPA Infection Committee) – April 2026**  Thank you for the opportunity to feedback on this guideline. This is an important, timely, and highly worthwhile piece of work addressing a clinically significant and preventable cause of harm. The guideline is grounded in expertise across genomics, infectious diseases, neonatology, pharmacy, and health economics, and it is particularly strong in bringing together the scientific rationale, clinical context, and implementation ambition for *MT-RNR1* testing in a way that is highly relevant to UK practice. Overall, it is a thoughtful and valuable draft that has the potential to make a meaningful contribution to safer aminoglycoside prescribing. We offer collated feedback on behalf of UKCPA specialist committees additionally extended to GMSA Pharmacy leads.  **Summary points**   - Overall there are a lot of very important and useful action recommendations in the document, but they are not always in the expected or consistent sections. - It would be helpful to tabulate or bullet point the testing recommendations and the clinical actions based on genotype for each indication/clinical scenario in which aminoglycosides are used (eg predicted/planned use, acute sepsis, acute non-sepsis, topical, otic, inhaled, vaccination etc). - Where SmPCs are quoted, the specific brand should be named in the reference section, as SmPCs are product-specific and not all SmPCs have identical text. - Routes other than systemic administration are discussed in the document, but the scope of the review and the resulting clinical recommendations is unclear for otic, ophthalmic, topical, inhaled and enteral products. This could be summarised more clearly and include route-stratified recommendations in section 7.   **Section 1: Background and overview**  On the incidence of ototoxicity, antimicrobial colleagues signposted to the following recent studies if helpful to provide more context e.g. “17% of patients develop ototoxic shifts after a course of IV aminoglycoside”.   - Incidence 17% (courses <16 days) and >40% in MDRTB:  Prasad K, Borre E, Dillard L et al.  Priorities for hearing loss prevention and estimates of global cause-specific burdens of hearing loss: a systematic rapid review. The Lancet Global Health, 12, e217-e225      - 17% in resp cohort: Cheong J, Boreland S, Shattock E, et al. Improving ototoxicity monitoring in patients receiving aminoglycosides using a novel digital approach: a quality improvement project. BMJ Open Quality 2025;14:e002847. doi:10.1136/bmjoq-2024-002847     In the last paragraph, consider also including mention of reflex testing for patients who have positive CF confirmatory genetic testing, which may be relevant when discussing the Newborn screening/ generation study sections. This is already routine practice in England via the GMS.    **Section 2 licensed indications**  There is a mix in this section between licenced indications and clinical uses and would benefit from clearer separation between licensed indications and broader clinical uses which may be better moved to Section 5.  Table “Current licenced indications for gentamicin in the UK”   - SmPCs are specific to a product (including formulation and strength) and not a drug. We suggest being specific which medicinal product SmPC this is from, since the different products have different wording of licensed indications. - The list provided looks like the specified indications from the SmPC for Gentamicin 40mg/ml solution for injection/infusion from Panpharma UK Ltd. It may be simpler to use the wording from the SmPC for the branded product Cidomycin 80mg/ml solution for injection which is “Gentamicin is indicated to treat severe infections caused by bacteria susceptible to gentamicin” and then if required provide some clinical use examples. - It is not clear from the table that other gentamicin preparations such as ear drops, and nebulised solutions are also available. - SmPCs can be referenced to directly via <https://www.medicines.org.uk/emc> .   See also comments on Appendix 1 below.  The UKCPA Infection committee also advise re: the following:  Quoting: “Gentamicin is no longer recommended..[endocarditis treatment]”: It is not recommended in MSSA native valve endocarditis but still recommended in prosthetic valve endocarditis for the 1st two weeks caused by MSSA and MRSA. See page 3980-1 in ESC IE guidelines. Also recommended for Strep IE in the shorter 2-week regimen, higher resistant Streptococcal species and enterococcal species (except in high-level aminoglycoside resistance). Regarding the section 2 NTM paragraph, “Also recommended in cystic fibrosis (as mentioned later in document) by CF trust.   - Consensus documents and American guidelines - Flume PA, Mogayzel PJ Jr, Robinson KA, Goss CH, Rosenblatt RL, Kuhn RJ, Marshall BC, Clinical Practice Guidelines for Pulmonary Therapies Committee. Cystic fibrosis pulmonary guidelines: treatment of pulmonary exacerbations. Am J Respir Crit Care Med. 2009 Nov 1;180(9):802-8. doi: 10.1164/rccm.200812-1845PP. Epub 2009 Sep 3.   Also recommended in non-CF bronchiectasis (as mentioned later in document) by BTS and ERS guidelines:   - Pasteur MC, Bilton D, Hill AT; British Thoracic Society Bronchiectasis non-CF Guideline Group. British Thoracic Society guideline for non-CF bronchiectasis. Thorax. 2010 Jul;65 Suppl 1:i1-58. doi: 10.1136/thx.2010.136119. PMID: 20627931. - Polverino E, Goeminne PC, McDonnell MJ, et al. European Respiratory Society guidelines for the management of adult bronchiectasis. Eur Respir J 2017; 50: 1700629. doi:10.1183/13993003.00629-2017   **Section 3 evidence overview**  Very useful recommendations around heteroplasmy. Can a reference be provided for the statement that “levels of heteroplasmy differ between different tissues and may not accurately reflect levels in the inner hair cells”? Should this be “inner hair cells in the cochlea?”  Consider discussing the increased risk in patients with co-morbidities such as renal impairment here instead of section 4.  Should the nuance that “any level of heteroplasmy” should be considered significant be included in section 7?  **Section 4 Recommended indications for PGx testing**  Agree with the recommendation to not delay aminoglycoside administration where clinical risk is severe and aminoglycosides are the preferred treatment.  Paragraph re: otic use – ‘relatively contraindicated’ may cause confusion - consider elaborating on ‘relatively’ or change to just ‘contraindicated’.  Could a brief explanation of what a level 1 investigation means be included – from British Association of Audiovestibular Physicians guidance – ‘....a level 1 investigation i,e, offered to all children with progressive...’  Does the bold summary sentence rule out testing for those receiving aminoglycoside ear drops where the tympanic membrane is intact?  Should there be clarification that patients receiving the drugs via enteral routes where there is epithelial damage will require testing in the summary sentence?  There is slight inconsistency within the routes of administration and recommendation for testing. The narrative discusses the risk of absorption with enteral in context of epithelial damage and inflammation, (which may be common in the conditions where enteral aminoglycosides are utilised). Consider rephrasing the exception to “Enteral and non-otic topical use are generally unlikely to require routine pre-treatment testing unless absorption is expected to be clinically significant”    The NICE HTG665 guidance recommends that *[1.3] Positive results [from Genedrive POCT] should be confirmed by laboratory testing*. Please consider the impact and role for this in the lab testing indication and in section 5 integrating in pathways.  **Section 5 integrating testing**  Overall, this section would be easier to read if it was divided into subsections for each of the indications for testing e.g. planned or predicted use of aminoglycosides, investigation of hearing loss, and acute sepsis/acute non-sepsis use. There are also some recommendations for indications for testing in this section, and some in section 6, so this could be made clearer by ensuring they are all in the same place.  Re: *“The use of aminoglycosides can be planned or predicted in about 20% of individuals”.* Please provide a reference for this statement.  It is recommended that in patients in whom the use of aminoglycosides can be planned or predicted “the nationally commissioned test in the National Genomic Test Directory‎ (NGTD) from NHSE should be requested”. This statement can only apply to patients treated in the NHS in England. As the guideline is likely to have wider impact (devolved nations, care outside the NHS) would it better to remove this statement and recommend instead testing for all 3 variants (or more if applicable). The indications listed under “these clinical scenarios include the following:” do not match the indications listed in the NGTD, which may create ambiguity although would be resolved if removing reference to the use of the NGTD.  Consider including “Or non-tuberculous mycobacterial pulmonary disease (NTM-PD)” in the third bulletpoint.  The market for POCT is growing. Another test has also recently been made available in the UK: <https://unahealth.co.uk/credo-vita-siro-solo-and-mt-rnr1-assay-achieve-ce-marking-a-breakthrough-for-rapid-life-saving-genetic-insight-in-neonatal-care/>  . Suggest not including details of any specific branded product but instead providing advisory of the expected standards and approvals for POCTs in the UK.  The guideline only covers “sepsis/suspected sepsis” as an acute use, so it is unclear what the recommendation is for acute non-sepsis indications such as infection without sepsis, catheter changes and surgical prophylaxis.  **Section 6 which genes, variants etc**  *“If in a setting where point of care testing is routinely delivered for MT-RNR1 variants and the test fails…….”* Suggest this recommendation be in Section 7 rather than section 6.  Recommendation to use a POCT in all patients with sepsis – if this is included consider also stating that this recommendation is dependent on there being evidence that doing so will not adversely affect the treatment pathway (as currently there is only evidence for this in NICU). Aminoglycosides are widely used in hospitals in almost all departments and so many POCTs are likely to be needed to be able to deliver this safely.  The UKCPA Critical Care Committee would support the recording of the genetic variation as a drug reaction in the allergy section in the medical record. If health economic benefit is established for pharmacogenetic testing in individuals in whom aminoglycosides can be planned or predicted, careful consideration needs to be given to how results for testing for MT-RNR1 are communicated in the patient’s record and available at the point of prescribing.    **Section 7 clinical actions based on genotype**  It is not clear if the recommendation to avoid aminoglycosides in individuals with a MT-RNR1 AIHL associated variant applies to all routes of administration, specifically to include inhaled, intraperitoneal, topical and otic routes. This is a question that comes to us (pharmacists) frequently. The SmPCs for some topical products (e.g. gentamicin ear drops) do include a warning regarding MTRNR1 variants although they don’t provide definitive recommendations on whether the product should be used. Although it is noted that in section 4 recommendations for testing that topical administration is unlikely to cause AIHL - it would be useful to then provide clear recommendations regarding clinical actions in this section, and with reference to the SmPCs for topical preparations if recommendations are not aligned with those in the product licenses.  It would be helpful to have a table, or bullet points with clear action-based statements in this section that specifically state “do not use” or “avoid” based on a positive result and with reference to all formulations/routes of administration that are available, including inhaled, topical, otic, vaccinations.  **Section 9 Economic evaluation**  The economic evaluation states that for cystic fibrosis (the only adult case stated), it’s not cost-effective; yet the guideline says this should be used – It would be useful if the authors further commented and to clarify the distinctions between the assumptions in this study and the guidance / UK pathway. For example, presumably, the different variants tested, allele frequencies, and the differences in antibacterial choices where “avoidance of the more effective first-line antibiotic therapy” could worsen outcomes in presence of MT-RNR1 variants.  **Section 10 regulatory considerations**  NICE has updated Health technology evaluation 6 (HTE6) to become [HealthTech guidance 665.](https://www.nice.org.uk/guidance/htg665)  The paragraph citing the PALOH-UK study should be moved to Section 12 (research recommendations) -it is not in keeping with the two other already published regulatory positions.  **Section 11 other considerations**  Given the increasing pressures on clinical genetic departments and efforts to mainstream discussions and testing pathways nationally, could the reference to referral to a Clinical Genetics Department be removed or re-phrased to encourage these discussions to be mainstreamed? e.g. “Routine pre-test counselling need not be extensive, but patients/parents/carers should be informed that a positive result may have implications for maternal relatives and future prescribing. This inheritance pattern can be explained through discussion with probands (patients directly tested) and their families following identification of a AIHL associated variant in MT-RNR1. Resources to support this discussion are available at GeNotes. Referral to clinical genetics or genomic counselling services should be considered where familial discussion or cascade implications are significant or in cases of uncertainty and other complexities”  Should the ‘other known health implications’ section discuss the equivocal evidence that having the variant in itself may cause SNHL in absence of aminoglycoside administration.  **Section 12: Research recommendations**  Is there an opportunity to assess how testing mothers in high risk pregnancy can infer MT-RNR1 genotypes in high risk births and neonates, thus providing an alternative option where POCT is unavailable.  More research is needed particularly on point of care genetic testing and the impact on prescribing pathways, before health economic benefits in non-NICU environments can be established.  **Appendix 1**  See also comments on Section 2   - SmPCs are specific to a product (including formulation and strength) and not a drug, so this should be made clear. It would be more consistent to standardise the wording in these tables to take the exact wording from the SmPCs (eg “XXX is indicated for......). - SmPCs can be referenced to directly via <https://www.medicines.org.uk/emc> - In some cases (e.g. amikacin) you have provided more detail about the drug beyond the licensed indication. Please revise some of the quoted wording and remove irrelevant/unnecessary information e.g. “surgical procedures should be performed.” - Amikacin (nebulised) is an additional formulation not reported in the appendix - <https://www.medicines.org.uk/emc/product/12067/smpc>  OR <https://bnf.nice.org.uk/drugs/amikacin/#indications-and-dose> - Provide a reference for the licensed indications for streptomycin and neomycin. - Streptomycin is not currently licensed in the UK (special order manufacturer only) so there is no UK SmPC. The table should be updated to reflect this (note the BNF <https://bnf.nice.org.uk/drugs/streptomycin/> specifically states “Use in tuberculosis is an unlicensed indication.” - Neomycin has licenced formulations for eye drops, ear drops and spray as well as ointments and creams (https://www.medicines.org.uk/emc/search?q=neomycin), mostly as combination preparations.  We are unsure which preparation is used for oral decontamination/SDD, is this an unlicenced import/indication? SmPC references should be provided here. - There are two other aminoglycosides licensed in the UK that have not been included in appendix 1: netilmicin and framycetin sulfate. Both of these drugs are in the BNF and their pages in the BNF include a warning about MT-RNR1 variants: https://bnf.nice.org.uk/drugs/framycetin-sulfate/  ; <https://bnf.nice.org.uk/drugs/netilmicin/>     Tobramycin has licensed formulation for inhalation/nebulisation for  chronic pseudomonas aeruginosa infection in patients with cystic fibrosis (e.g. see SmPCs for Bramitob or Vantobra).    **Minor editing corrections**   - Please keep pharmacogenetic/genomic language terms consistent throughout the document - Page 4 – Actinomycetes should be capitalised and the comma removed (as other non-Actinomycete filamentous bacteria exist) - Page 5, missing the word “nationally” before “commissioned”. - Page 7. Paragraph 4: Consider rewording e.g. NICE-recommend**ed** treatment of sepsis or suspected sepsis in neonates is a combination of etc etc or NICE recommend treatment of sepsis in neonates **with** a combination of etc etc - Page 8 is “warranted” intentional or should it be “recommended” - Page 12, capitalisation of Dutch Pharmacogenomics Working Group in section heading - Review dates and versions of some references e.g. NGTD update to latest version, NICE HTE6 to HTG665 - CPIC MTRNR1 guidance is duplicated in references. - Check minor spelling errors e.g. tobyramycin | We thank the UKCPA for the positive comments and we have responded to the comments individually, below.  We recognise and accept the comment. However, the guideline is presented in the same format as other CERSI-PGx guidelines, and we believe that this consistency has merits beyond differing formats.  We have amended the text to recognise this important point.  We have amended to ensure that this is clear. As stated in the summary statement at the end of section 4, MT-RNR1 testing is recommended “where this is possible in a clinically achievable time frame prior to use for all aminoglycosides and for all routes of administration, except topical or enteral.” This has also been reinforced at start of section 7.  We thank the reviewer for highlighting these useful references which have been included within the guideline.  We have added a note regarding reflex testing to section 5.  We have amended the text regarding the SmPC recognising that this is a single product specific exemplar.  We have amended the text in response to this comment.  We note that the recommendation for use of gentamicin is captured in the SmPC in the context of cystic fibrosis and bronchopulmonary infections. The text is to provide some additional context and so we have not amended this section.  No specific reference is available for MT-RNR1 as this is fundamentally true of heteroplasmy where levels of mitochondrially encoded variants are different in different tissues.  Inner hair cells in the cochlea is now made explicit in the text.  We have moved the recommendation to section 7 as suggested  We have removed the text ‘relatively’ as suggested.  A broader explanation of testing to investigate hearing loss is outside the scope of this guideline and can be referred to in the reference.  We have revised the text in the bold-paragraph to include ‘(unless absorption is expected to be clinically significant).’  We have amended the text to make clearer regarding routes of administration guidance  This is not an indication for pharmacogenetic testing – it relates to confirmation of a result and so we feel does not sit in section 4. It is covered in section 10.  We understand and appreciate the comment but have aligned the format with previous CERSI guidance  There is not a reference for this statement as it is based on real time hospital data.  We have made it clear throughout the guideline that the NGTD is only relevant to England.  Our guidance recommends testing of MT-RNR1 variants when aminoglycoside testing is planned.  The NGTD guidance is not exhaustive but provides key exemplars where such testing is clinically indicated prior to planned aminoglycoside use.  We have included an additional bullet point to this section **‘individuals with non-tuberculous mycobacterial infection e.g. *M.avium, M.abscessus*.’**  We recognise that other tests are and will become available, however at the time of writing only one test as stated has been through NICE evaluation and has generated published evidence in neonatal units England. Therefore, we comment on this test in the guideline.  As the guideline is updated it will be appropriate to add further information.  We agree with the commnet and have moved this paragraph to Section 7 as suggested.  We have amended this section to reflect the comment and also included a recommendation for consideration of point of care testing in other acute settings being evaluated by further research.  We thank the reviewers for the important comment.  The text is made explicit for all routes of administration except topical or enteral (unless clinical evidence suggesting higher levels of absorption)  This has been amended  We understand and appreciate the comment but have aligned the format with previous CERSI guidance  It is difficult to extrapolate from this USA study. Further analyses specific to relevant health care settings are required.  We have updated the NICE HealthTech guidance to HTG665, as suggested.  If neonatal testing was introduced in England this would result in ~10 referrals per year per Clinical Genomics Service (<1 per month). The value of this information being cascaded to the family outweighs the burden to clinical services. Mixed models should be explored for disseminating this information.  It is not equivocal and so the statement remains that “there are no other known health-implications of a MT-RNR1 variant that require any pre-test counselling”  We have updated the text to reflect these points.  We have amended these references as suggested.  We thank the reviewers for this comment. The text has been taken as stated in full from the SmPC and so has not been amended.  We are not providing an exhaustive list of all aminoglycoside products as that is not the purpose of this guideline but not that different formulations and products are available.  The appendix has been altered in light of these helpful comments  As suggested, this has been standardised throughout the guideline.  As suggested, Actinomycetes has been capitalised and comma removed.  As suggested, the word ‘nationally’ has been included.  As suggested, this paragraph has been reworded.  As suggested, the Dutch Pharmacogenomics Working Group has been capitalised.  As suggested, the NICE HealthTech guidance has been updated to HTG665 and the National Genomic Test Directory v9 has been referenced.  As suggested, the duplicate CPIC reference has been removed.  We have corrected the minor spelling error. | No  Yes, section 2, reference list and appendix 1  Yes, section 7  Yes, section 1 and reference list  Yes, section 5  Yes, section 2, appendix 1 and reference list  Yes, appendix 1 and reference list  No  No  Yes, section 7  Yes, section 7  Yes, section 4  No  Yes, section 4  Yes, section 4  No  No  No  No  No  Yes, section 5  No  No  Yes, section 6 and 7  Yes, section 6 and 12  No  Yes, section 7  Yes, section 7  No  No  Yes, section 10, reference list and appendix 1.  No  No  Yes, section 12  Yes, appendix 1 and reference list  No  No  Yes, appendix 1  Yes, throughout sections  Yes, section 1  Yes, section 1  Yes, section 4  Yes, section 8  Yes, section 10, reference list and appendix 1.  Yes, reference list.  Yes, reference  list. |
| Royal Pharmaceutical Society | No response received |  |  |
| National Pharmacogenomics Group (NHS Wales) | No response received |  |  |
| Royal College of Paediatrics and Child Health (RCPCH) | No response received |  |  |
| British Society for Antimicrobial Chemotherapy (BSAC) | No response received |  |  |
| Bliss | No response received |  |  |
| National Deaf Children’s Society (NDCS) | No response received |  |  |
| Royal National Institute for Deaf People (RNID) | Thank you for inviting RNID to comment on the guidelines you have developed for MT-RNR1 genotyping before prescribing aminoglycosides.  Whilst we are not able to comment on the technical or clinical aspects of the guidelines, we welcome efforts to improve and standardise clinical guidelines to reduce the risk of ototoxicity caused by aminoglycosides.  We fully support the recommendations that genetic testing of MT-RNR1 should be offered to all patients prior to aminoglycoside use where this can be achieved in a timeframe that does not impact on clinical outcomes. The roll out of rapid point of care tests is vital to ensuring timely genetic information can also be provided in acute care settings.  Hearing loss caused by aminoglycosides is irreversible and can significantly impact on quality of life. Clear and effective guidance on MT-RNR1 pharmacogenetic testing can help reduce the incidence of this avoidable cause of hearing loss. | Thank you for the positive comments and support. |  |
| The Association of the British Pharmaceutical Industry (ABPI) | No response received |  |  |
| British In Vitro Diagnostics Association (BIVDA) | I have read the document provided in detail and I have a couple of questions:   1. Given that in 2023 NICE (in the published early value assessment considering the use of point of care testing in an acute neonatal setting in the context of sepsis or suspected sepsis) made the following recommendations: Positive point-of-care results should be confirmed by laboratory testing. How long does it take for a confirmatory test and does this delay the treatment? 2. I understand the fail rate of GeneDrive test is 5%. What is the sensitivity and specificity of the test and should this be quoted in the paper? | Thank you, in response to the questions:  Treatment decisions are made on the basis of the point of care test. The confirmation testing in the laboratory is to determine the specificity of the test (number of false positive results). This information is important for future antibiotic treatment in the individual and appropriate counselling of family members.  The PALOH-UK study (ongoing and due to report at the end of 2026) is systematically assessing the sensitivity and specificity of the test | No  No |
| ClinPGx | No response received |  |  |
| Dutch Pharmacogenetics Working Group (DPWG) | No response received |  |  |
| British Infection Association (BIA) | Many thanks for consulting the British Infection Association on Guideline on MT-RNR1 genotype testing for preventing aminoglycoside-mediated ototoxicity.    In my role as guidelines secretary for BIA, I have reviewed the manuscript and provided some comments. These comments should be considered as advisory. I am afraid that in accordance with BIA practice, we cannot offer specific endorsement of these guidelines at this stage as the association was not involved with their development from inception.    I have included detailed comments in the attached version of the manuscript and summarised here below:     - Clarify how this guideline document relate to existing UK guidance.   - Is it about operationalisation of existing recommendations for MT-RNR1 testing or does it advocate extending/modifying recommendations?   - Similarly, does it complement or supersede any previously published guidelines, notably the Clinical Pharmacogenetics Implementation Consortium Guideline for the Use of Aminoglycosides Based on MT-RNR1 Genotype (DOI: 10.1002/cpt.2309).   - As I understand it, in advocating for the use of Point-of-care testing in all settings where aminoglycosides are used acutely, the recommendations in the current manuscript go beyond the conditional recommendations in NICE Early Value Assessment.      - Some clarification regarding guideline development methodology would be helpful.   - In section 3 - Evidence Overview - it would be helpful to include here a summary of your evidence search strategy so readers can make an assessment that all relevant available evidence has been captured and evaluated.   - It would be helpful to include details of how these recommendations have been formulated. For example using GRADE methodology or alternative Modified DELPHI process or other methodologies for consensus-finding?   - Can you include details of how this economic evaluation (in section 9) has informed/influenced your recommendations regarding testing.      - Suggest that all specific guideline statements made in this paper are compiled into a 'Summary of Recommendations' section with some comment on strength of recommendations and certainty of evidence supporting recommendation.      - Based on performance of currently available tests (including both point-of-care and lab-based assays) and considerations of sensitivity based on heteroplasmy, can you present estimates of positive predictive values and number needed to test to avoid harm under a range of plausible scenarios.      - Regarding indications and relevant patient groups:   - In view of high rates of ESBL, use of an initial dose of aminoglycoside is common practice in treating adults with suspected sepsis of unknown origin. Consider recommendations given widespread use in this context.   - Prolonged aminoglycoside use for the treatment of MDR-TB is now fairly unusual in UK practice given availability of effective oral agents (e.g. bedaquiline, linezolid etc). Aminoglycosides might be used in specific contexts e.g. severe disease, oral route temporarily not available, other toxicities/co-morbidities (e.g. DILI, Liver disease) limiting other treatment options.   - They are increasingly used in the management of non-tuberculous mycobacterial infection and nebulised liposomal amikacin (Arikayce) has now been licensed for the treatment of refractory Mycobacterium avium pulmonary disease and available via NHSE commissioning criteria with typical treatment durations of 6 months. Consider whether degree of systemic amikacin exposure is deemed significant with Arikayce to warrant testing here.      - For a failed point of care test, is the recommendation here to repeat the point of care test or to send a laboratory-based test to NHS Genomic Testing lab. If the latter, given the turn around time, most patients with a failed test would then be committed to an aminoglycoside sparing regimen, limiting therapeutic options.     **Comment in the text:**  ***Abstract***  How does this guideline document relate to existing UK guidance. Is it about operationalisation of existing recommendations for MT-RNR1 testing or does advocate extending/modifying recommendations?  Suggest that all specific guideline statements made in this paper are compiled into a 'Summary of Recommendations' section following the abstract.  **Section 1. BACKGROUND and OVERVIEW**  With the recent availability of highly effective oral agents developed/assessed for the treatment of MDR-TB, aminoglycosides are less frequently required. They have still have a role in extensive drug resistance or where oral treatment is not possible (e.g. critical care).  They are increasingly used in the management of non-tuberculous mycobacterial infection (e.g. M.avium, M.abscessus).  **Section 2. LICENSED INDICATIONS**  ESC guidelines still recommends the use of gentamicin for prosthetic valve endocarditis due to staphylococcal species.  **Section 3. EVIDENCE OVERVIEW**  In terms of guideline methodology, it would be helpful to include here a summary of your evidence search strategy so readers can make an assessment that all relevant available evidence has been captured and evaluated.  What extent of exposure? Single dose? Prolonged course?  Based on performance of current tests - including both point-of-care and lab-based assays - and these considerations of sensitivity based on heteroplasmy, can you present estimates of positive predictive values and number needed to test to avoid harm under a range of scenarios.  This could then be linked to cost-effectiveness estimates considered in later sections.    **Section 4. RECOMMENDED INDICATIONS FOR PHARMACOGENETIC TESTING**  It terms of guideline methodology, it would be helpful to include details of how these recommendations have been formulated. GRADE? Modified DELPHI or other methodologies for consensus-finding?  This is also common practice for adults with suspected sepsis of unknown origin - particularly in view of increasing rates of ESBLs.  Nebulised liposomal amikacin (Arikayce) has now been licensed for the treatment of refractory Mycobacterium avium pulmonary disease. Typical treatment duration is 6 months. Is there a specific recommendation for this form?  typo - add space  **Section 5. INTEGRATING PHARMACOGENETIC TESTING INTO EXISTING CLINICAL PATHWAYS**  See earlier comment - prolonged aminoglycoside use for MDR-TB is now unusual in UK practice. Aminoglycosides might be used short term in specific contexts e.g. severe disease, oral route temporarily not available, other toxicities/co-morbidities (e.g. DILI, Liver disease) limiting other treatment options.  If degree of systemic exposure is deemed significant with Arikayce, then it might be a good idea to include within the commissioning criteria to access Arikayce - currently done via BlueTeq as high cost drug.  **Section 6. WHICH GENE(S), VARIANTS, TURNAROUND TIME?**  Or adult? Aminoglycoside use is widespread in this context for adults also.  Is the recommendation here to repeat the point of care test or to send a laboratory based test to NHS Genomic Testing lab. IF the latter, given the turn around time, most patients with a failed test would then be committed to a aminoglycoside sparing regimen, limiting therapeutic options.  **Section 7. CLINICAL ACTIONS BASED ON GENOTYPE**  ?Formulate along the lines of GRADE recommendations or similar - conveying certainty of evidence and strength of recommendations.  Referring to ***The Clinical Pharmacogenetics Implementation Consortium guideline:***  Does this current guidelines document supersede these recommendations?  **Section 9. ECONOMIC EVALUATION**  Can you include details of how this economic evaluation has informed/influenced your recommendations regarding testing.  See earlier comment about including details of search strategy earlier in document in methodologies section.  ***10.2 NICE Early Value Assessment [HTE6]***  But as I understand it, this current manuscript in sections 4 and 5 goes beyond this conditional recommendation to advocate for the use of Point-of-care testing in all settings where aminoglycosides use acutely. | Thank you for the helpful comments these have been incorporated into the updated draft  This guideline provides clinical and UK specific context to previous guidance noted in section 8.  This guideline does not supersede previous guidelines.  This has been amended.  We appreciate this point but have set out the guideline in accord with the other CERSI-PGx guidelines. The methodology is included in the supplement. The recommendations are based on consensus based on extensive literature evaluation and discussions. We do not use GRADE methodology.  We recognise this point but have set out the paper in the format of the other CERSI-PGx guidelines  It will be appropriate to present this data in an update following the publication of the PALOH-UK study of 5550 individuals in 2026.  We have amended the text to reflect these important points.  We have amended the text to recognise this comment.  Note previous response.  Note previous response.  We have amended the text to reflect this comment.  As suggested, the text in this section has been amended to clarify this.  Note previous response.  Previous studies are mixed and so we have reflected this. We note that ototoxicity can occur after a single exposure.  Note previous response.  Note previous response.  We have noted in amended text.  We note that testing is indicated for all administrations except topical and enteral.  As suggested, the text has been amended.  Comment noted.  We have amended the text.  Note previous response.  Note previous response.  This guideline does not supersede previous guidelines.  Health economic analysis as part of PALOH-UK is ongoing and will be published shortly.  Note previous response.  Note previous response. | No  No  Yes, section 6  No  No  No  Yes, section 5.  Yes, section 7.  No  No  Yes, section 5.  Yes, section 2.  No.  No.  No.  No.  Yes, section 4.  No.  Yes, section 4.  No.  Yes, section 6.  Yes, section 7.  No.  No.  No.  No.  Yes, section 5 and 6 |
| Hospital Infection Society | No response received |  |  |
| Infection Prevention Society | No response received |  |  |
| Scottish Antimicrobial Prescribing Group (SAPG) | No response received |  |  |
| Neonatal & Paediatric Pharmacy Group (NPPG) | Thank you for providing NPPG the opportunity to review the CERSI-PGx guideline for MT-RNR1 genotyping with aminoglycosides. Comments from our committee members are as follows:  Page 4: states ‘In 1993, a variant m.1555A>G in the mitochondrial DNA encoded gene MT-RNR1 (MT-RNR1) was associated…’ – presume this should be ‘…was found to be associated…’.  Page 5 indicates that the guideline will focus on gentamicin as the most frequently used aminoglycoside in the UK. However, some neonatal units use amikacin first line for neonatal sepsis. Could this be acknowledged?  Page 5: The list of licensed indications is from a specific gentamicin product, but it does not state which one. The reference to the SmPC (ref 8) links to the MHRA search page, rather than to a specific SmPC. If licensed indications are to be included, I think it should be a comprehensive list of all licensed gentamicin products (or e.g. all licensed parenteral products), with off-label uses listed separately.  Page 6: states ‘Gentamicin should be given as a single daily dose…’ – please acknowledge that neonatal dosing may be initiated at 36 hourly intervals.  Page 6: The term ‘penetrance’ may need explanation for a non-specialist audience (e.g. in a glossary? Or in brackets after the word, as for heteroplasmy in the paragraph below?)  Page 10: states ‘e.g. suspected sepsis or sepsis in a neonate, when the decision to use an aminoglycoside is time-sensitive, a point of care test is recommended’, whereas most other parts of the document state to use a point of care test in acute setting if available. I am worried that making a clear recommendation opens centres up to litigation if they do not have point of care testing, so I would suggest a rewording (or more careful) wording of this section.  Page 10: Suggest a comment is added to emphasise that POC testing may not identify a relevant variant that would be picked up by laboratory testing. GeneDrive is described as a ‘LAMP-based assay’ but there is no explanation about what this is. Is this detail needed?  Page 10: states that if test failure occurs the non-aminoglycoside pathway should be followed. Is there a reference for this recommendation? Interested to understand why the recommendation is different to settings where POC testing is not available.  Page 11: could the recommendation to avoid aminoglycosides confirm whether or not this includes topical and enteral administration.  Page 11: states the reasons why the absence of a MT-RNR1 AIHL associated variant does not eliminate the risk of AIHL, but does not mention that POC testing does not currently cover all known causal variants – this is an important acknowledgement, particularly within a neonatal setting where POC testing may occur.  Page 14: the familial implications are described as if the mother is the index case. It may be helpful to provide an example to clarify the consequences for a child’s siblings (e.g. if a neonate is found to have a variant).  Also:  With regards to point of care testing – the current situation is inequitable as it is mostly the centres involved in the current PALOH-UK study and a few individual centres that have self funded. Not sure if the working group feel happy to highlight this and make a recommendation that if NICE review the PALOH-UK data and feel that they can make a positive opinion of this, that centralised funding should be made available.  As a thought for the working group, should there be any consideration of the lab test for the pregnant mother in a ‘high risk’ pregnancy. This might identify neonates that may possibly have the gene variant and suggest they avoid aminoglycosides until full testing of the neonate is possible? | Thank you for the helpful comments these have been incorporated into the updated draft.  Noted.  Gentamicin is recommended by NICE [NG195] and so we have not explicitly noted the use of amikacin.  As suggested, we have amended the text regarding the SmPC recognising that this is a single product specific exemplar.  As suggested, the text has been amended to reflect this.  The text **“(development of SNHL in individuals harbouring a pathogenic *MT-RNR1* variant following exposure to an aminoglycoside)”** is included in this section to explain the term.  As suggested, this paragraph has been updated to more clearly capture the option of a point of care test.  As suggested, the description of the genedrive MT-RNR1 test has been updated to provide clarity.  We have amended this section to reflect that local microbiology guidance should be followed, with the following added: **‘If in a setting where point of care testing is routinely delivered for MT-RNR1 variants and the test fails (fail rate is <5%), then if an antibiotic is required before a repeat test can be undertaken, the antibiotic pathway (as dictated by local microbiology guidance) should be used’**  As suggested, we have added further clarification to this section.  We have reworded this section to address the comments.  As suggested, this has been amended to add further examples.  We acknowledge this point but do not feel it fits within a guideline but have been in discussion with NICE and the NHS Commissioning teams to address.  This is a consideration and can be undertaken but requires testing in practice to see if the information is transferred to inform neonatal antibiotic prescribing. Added to research section. | No  No  Yes, section 2  Yes, section 2  No  Yes, section 6  Yes, section 6  Yes, section 6  Yes, section 7  Yes, section 7  Yes, section 11  No  Yes, section 12 |
